# Feline disseminated cutaneous phaeohyphomycosis due to *Exophiala spinifera*

**DOI:** 10.1016/j.mmcr.2019.12.008

**Published:** 2019-12-24

**Authors:** Julie-Anne Daly, Vit Hubka, Alena Kubátová, Marina Gimeno, Vanessa R. Barrs

**Affiliations:** a University of Sydney, Faculty of Science, Sydney School of Veterinary Science, Camperdown, NSW, 2050, Australia; b Department of Botany, Faculty of Science, Charles University, Benátská 2, 128 01, Prague 2, Czech Republic; c Laboratory of Fungal Genetics and Metabolism, Institute of Microbiology of the Czech Academy of Sciences, V. v. i., Vídeňská 1083, 142 20, Prague 4, Czech Republic; d University of Sydney, Faculty of Science, Sydney School of Veterinary Science, Camden, NSW, 2570, Australia

**Keywords:** Black yeasts, *Exophiala*, Phaehyphomycosis, Histopathology, Molecular diagnosis

## Abstract

A six-year-old domestic shorthair cat was presented for a subcutaneous digital nodular lesion on the right forelimb. On physical examination a similar lesion was identified on the right hindlimb. Disseminated cutaneous phaeohyphomycosis was diagnosed from histopathological changes in representative tissue biopsies and fungal culture. The isolate was identified by sequencing of ITS rDNA as *Exophiala spinifera.* This is the first report of disseminated cutaneous disease caused by *E. spinifera* in the cat.

## 1 Introduction

Multiple genera of melanin-pigmented dematiaceous saprophytic fungi can cause opportunistic fungal infections (phaeohyphomycoses) in humans and animals, usually by traumatic cutaneous inoculation, for example by splinters or thorns [1,2]. Phaeohyphomycoses are uncommon in cats and are typically characterised by cutaneous or subcutaneous lesions affecting the nasal planum or digits, although focal invasive and disseminated infections also occur [1,3,4]. The most common fungal genera implicated in feline phaeohyphomycoses include *Alternaria, Cladophialophora, Microsphaeropsis* and *Exophilia* [3,[5], [6], [7]]*. Alternaria* and *Cladophialophora* spp. are also the most frequent fungal organisms detected on feline skin [8]. Regional differences in the prevalence of the causal agents of feline phaeohyphomycoses appear to occur, for example in the UK and mainland Europe *Alternaria* spp. are the most frequent cause, while in Australia *Microsphaeropsis arundinis* is detected most frequently [[3], [4], [5]].

Due to the traumatic mode of inoculation, cutaneous lesions in phaeohyphomycoses are usually singular. *Exophiala* spp. have been detected in 10 cases of feline phaeohyphomycosis, of which nine were associated with solitary cutaneous or mucocutaneous lesions affecting a limb, the nasal cavity and/or nasal planum, or face [4,6,7,[9], [10], [11], [12], [13], [14]]. Three species in the genus, *E. attenuata* (n = 2)*, E. jeanselmei* (n = 4) and *E. spinifera* (n = 3) are associated with disease in cats.

This is the first report of disseminated cutaneous disease caused by *E. spinifera* in a cat. Three other cases of phaeohyphomycosis caused by this species have been described in cats but only one of these was definitely identified using molecular sequencing [15].

## 2 Case

**Fig. 1:**
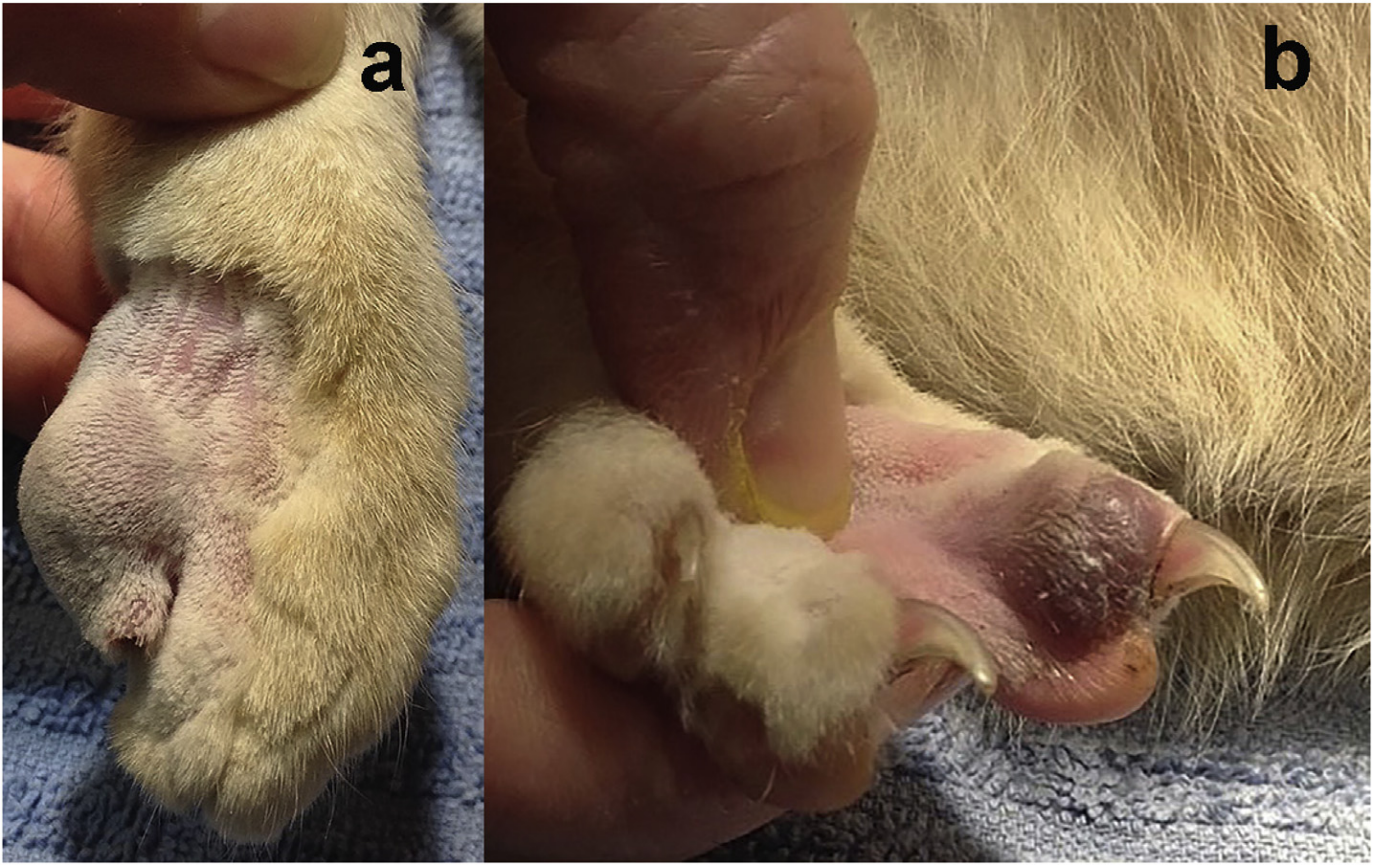
Digital subcutaneous nodules on (a) the distal 5th phalanx (P5) of the right forelimb and (b) the distal 3rd phalanx (P3) of the right hindlimb.

A 6-year old male neutered domestic shorthair cat was presented by its owner for investigation of a nodular digital swelling affecting the right forelimb (RFL) (Day 0). The cat had indoor and outdoor access. It had been presented a year ago for a similar subcutaneous mass on the third distal phalanx (P3) of the right hindlimb (RHL), which had been diagnosed on fine-needle aspiration cytology as a fungal granuloma containing yeast and hyphal forms. The mass had been resected but the infecting fungal species was not identified. On physical examination on Day 0 a 10 mm diameter, discrete, focally extensive, subcutaneous, non-pigmented nodule was identified on the distal P5 of the RFL, which was non-painful on palpation (Fig. 1 a). A second, focally extensive, purplish subcutaneous 5 mm nodule was identified proximal to the nailbed on the distal P3 of the RHL (Fig. 1 b). Physical examination was otherwise unremarkable, and all vital signs were normal.

Results of a complete blood count, and total thyroxine measurement were within reference intervals, and serology for Feline immunodeficiency virus (FIV) antibody and Feline leukaemia virus (FeLV) antigen (IDEXX-SNAP, Pty Ltd) was negative. Abnormalities on a serum biochemical profile included a mild azotaemia (11.2 mmol/L; reference range 3.0–10.0mmol/L), mild elevation in creatine kinase (454U/L; reference range <261U/L), and a mild hypertriglyceridaemia (0.8mmol/L; reference range 0.1–0.6mmol/L). Fine-needle aspiration cytology of the lesions showed the presence of neutrophils and numerous macrophages, some containing fungal hyphae and yeast forms. Punch biopsies of the lesions were collected under general anaesthesia on Day 14 for fungal culture and histopathology. Pending results, the cat was commenced on treatment with 50 mg of itraconazole and 62.5 mg of terbinafine orally, once daily. The owner discontinued the terbinafine after four days administration, and then discontinued itraconazole two days later, due to episodes of vomiting. Posaconazole therapy was recommended and declined. Three months after presentation (Day 101) the owner elected to euthanase the cat because the RHL lesion became ulcerated and bled, and the cat was lethargic. A post-mortem examination was not performed.

**Fig. 2:**
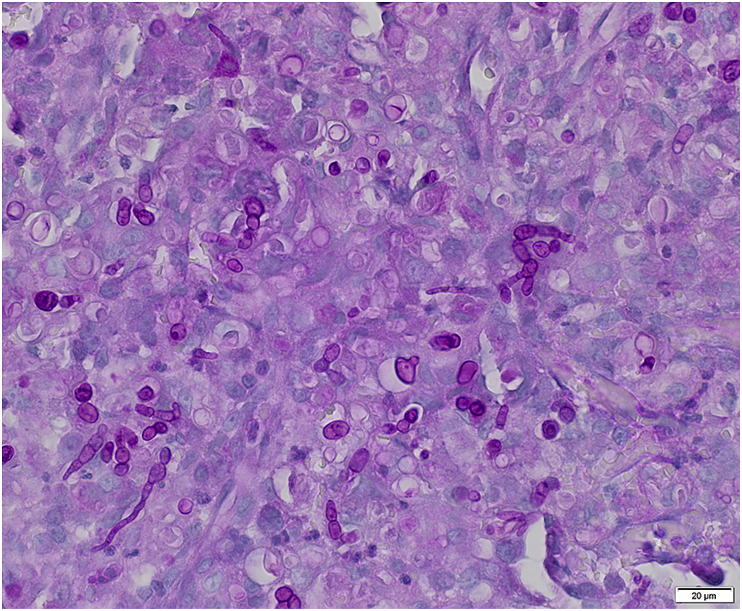
Ovoid yeast forms and non-parallel hyphae are markedly PAS-positive. Large amounts of pyogranulomatous inflammatory infiltrate efface the dermis. Periodic-Acid Schiff (PAS). Scale bar 20 μ

Histopathology sections of the biopsied digital masses, stained with hematoxylin and eosin, revealed diffuse, severe, pyogranulomatous dermal inflammation with intralesional pigmented yeast and hyphal forms. The dermis and deep dermis were expanded by multiple coalescing nodules of inflammation, composed of numerous viable and degenerate neutrophils, epithelioid macrophages, scattered multinucleated giant cells, and rare lymphocytes and plasma cells admixed with moderate numbers of reactive fibroblasts, eosinophilic cellular and necrotic debris and fibrin. Numerous pigmented yeast and hyphal forms were scattered throughout the nodules, including within the cytoplasm of macrophages and giant cells. Fungal elements were markedly positive for Periodic-Acid Schiff (PAS) stain. Yeasts were ovoid, 7–20 μm in diameter, with 2–3 μm-thick golden cell walls, clear to pale cytoplasm with a central basophilic nucleus. Hyphae were 5–10 μm wide, septate with irregular, branched or unbranched with non-parallel pigmented walls (Fig. 2).

Black yeast-like colonies were isolated from both digital lesions on malt-extract agar (MEA) at 37 °C. Subcultures of the case isolate were grown on MEA and potato–carrot agar (PCA) at 25 and 37 °C in the dark. The isolate was deposited into the Culture Collection of Fungi (CCF) at the Department of Botany, Charles University, Czech Republic under the number CCF 5820.

**Fig. 3:**
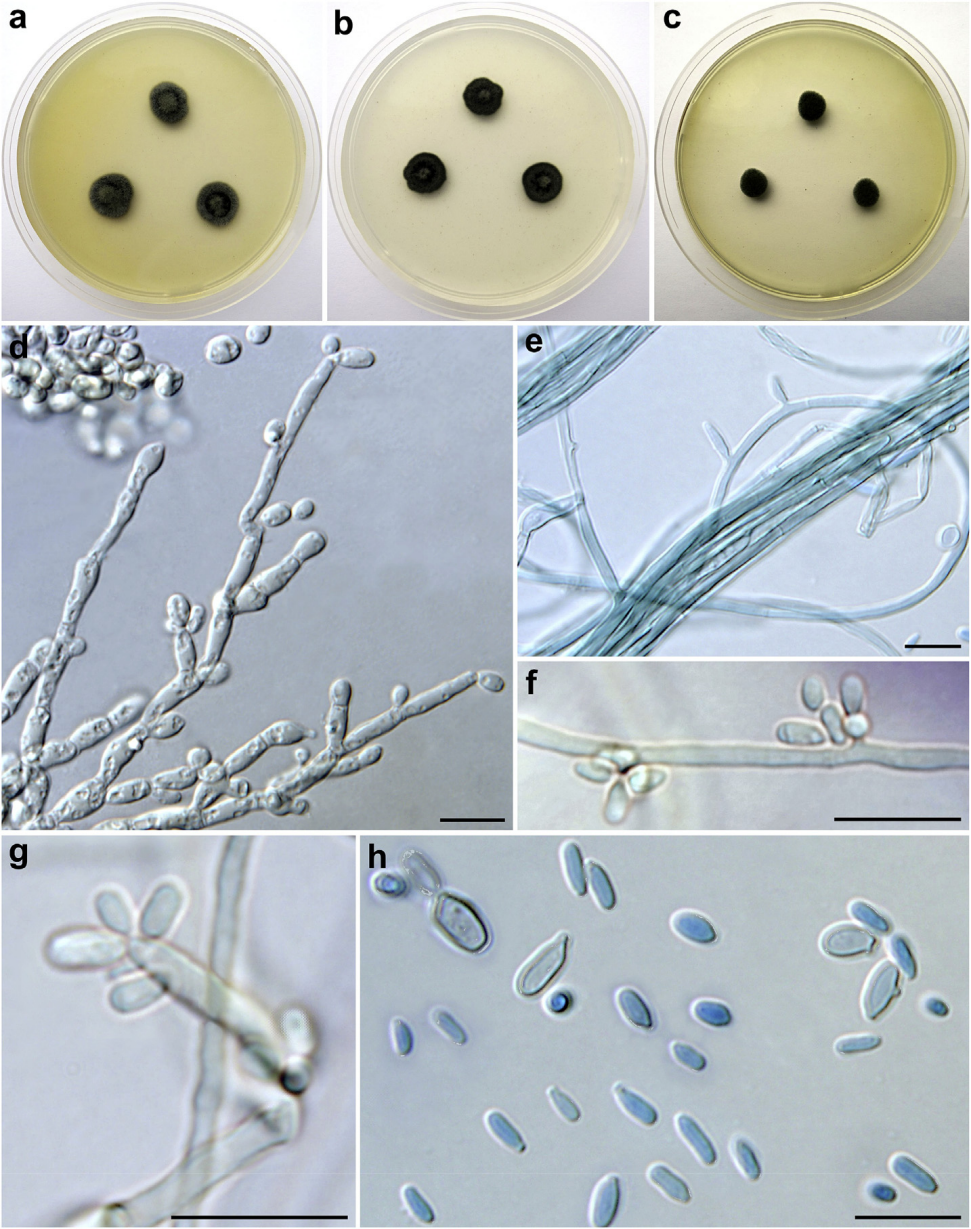
Macromorphology and micromorphology of *Exophiala spinifera* CCF 5820. Colonies incubated 7 days at 25 °C on MEA (a) and PCA (b); colonies on MEA at 37 °C after 7 days (c); poorly differentiated semi-micronematous or micronematous conidiophores producing conidia at apical or lateral conidiogenous loci (d-g); free conidia (h), a budding yeast-like cell is visible in the left corner, two cells with lateral bud scar are present in the right corner of the subfigure. Scale bars: 10 μm. For micromorphology lactic acid with cotton blue was used as a mounting medium. (For interpretation of the references to colour in this figure legend, the reader is referred to the Web version of this article.)

Colonies on MEA at 25 °C (Fig. 3a) attained 10–13 mm diameter after 7 days (20–23 mm after 14 days); lanose; raised; olive (3F5) to olive grey (3F2); reverse dark greyish brown (5F3); at 37 °C 3–5 mm diameter after 7 days (Fig. 3c) (8–10 mm after 14 days). Colonies on PCA at 25 °C (Fig. 2b) attained 10–12 mm diameter after 7 days (17–19 mm after 14 days); velvety to lanose; umbonate; dull green (30E3) to dark green (30F5); reverse dark green (27F3); at 37 °C 12–15 mm diameter after 7 days (17–20 mm after 14 days).

Microscopic examination revealed the presence of masses of one-celled, smooth-walled, hyaline to mid-brown, ovate or ellipsoidal conidia, mostly 3–5(‒5.5) × 1–2 μm (4 ± 0.6 × 1.5 ± 0.2; mean ± standard deviation), sometimes blastically budding at terminal or lateral positions (Fig. 2h). Conidiophores were usually poorly differentiated from vegetative hyphae (Fig. 3d–g), micronematous to semi-micronematous; conidia arising alongside the hyphae at the intercalary conidiogenous loci or at the apices of poorly differentiated conidiophores.

### 2.1 Molecular studies

Genomic DNA was extracted from 7-day-old colonies grown on MEA (Oxoid Ltd., Basingstoke, UK) using the ArchivePure DNA yeast and Gram2 + kit (5PRIME Inc., Gaithersburg, MD, U.S.A.) according to the updated manufacturer's instructions [16]. The ITS rDNA region was amplified and sequenced as described previously [16].

Using the BLAST similarity search, the ITS region of the case isolate shared 98.7% identical base pairs (533/540 bp; seven substitutions, one insertion) with the ex-type strain of *E. spinifera* CBS 899.68 (AY156976); the next most similar species was *E. exophialae (*ex-type CBS 668.76; AY156973) with 96.3% similarity (522/542 bp; 14 substitutions, six indels); other *Exophiala* spp. showed similarity lower than 95%. The obtained sequence was deposited into the ENA (European Nucleotide Archive) database under the accession number LR594658.

## 3 Discussion

Our report is the first to describe more than one cutaneous/subcutaneous lesion in a cat caused by *E. spinifera* or by any other *Exophiala* spp. However, in humans with *E. spinifera* infections multiple cutaneous lesions are common, comprising primarily of a few lesions with regional spread or of few to multiple disseminated lesions [17]. Also, the majority of haematogenously disseminated dematiaceous yeast infections in humans are caused by *Exophiala* species, which are able to produce yeast-like cells. The ability of these opportunistic pathogens to bud facilitates haematogenous dissemination, whereas with hyphal growth alone, localized infection is more likely [18]. Immunosuppressive therapy is a predisposing factor for the development of multiple skin lesions in humans infected with *E. spinifera* and *CARD9* gene mutations are also suspected, which are associated with defective innate immune recognition of fungi, especially *Candida* and dermatophytes [17]. The cat in this report did not have any known co-morbidities and tested negative for FIV and FeLV, two common viral causes of immunosuppression in cats.

Including the case we have described here, three of the four cases of feline phaeohyphomycosis caused by *E. spinifera* were identified in Australia, and the other was from a cat in France [6,7]. Of approximately 40 cases of *E. spinifera* infection diagnosed in humans, most were also from tropical or subtropical regions and only one case of infection was diagnosed in Europe [17]. In general, members of the genus *Exophiala* are found commonly in soil, plant materials, extreme environments, hydrocarbon-polluted environments and indoor environments [19]. Species from the *E. spinifera* complex have a cosmopolitan distribution and are amongst the most aggressive pathogenic species of the genus [15].

Inoculation of one of the limb digits via trauma was the most likely route of infection of the cat described here, given the ubiquitous nature of *Exophiala* spp, with subsequent haematogenous dissemination. Since a post-mortem examination was not performed, we could not ascertain whether dissemination was more widespread.

The history of a previously excised digital fungal mass involving a different digit of the RFL in the case presented here may have also involved the same organism, although the identity of the isolate was not confirmed. Regional relapse of *E. spinifera* after excision has been reported in human infections [17].

Since microscopic morphology alone is insufficient to allow routine laboratory identification of black pigmented yeasts, molecular diagnostic techniques are necessary to confirm identification of the infecting agent. Reliable identification to a species level may also be important in understanding progression of the disease and response to treatment [15]. Although molecular characterisation of fungal pathogens is becoming more routine in veterinary medicine, few of the infecting isolates in previous reports of feline phaeohyphomycosis have been definitively identified using molecular techniques. The previous three cases due to *E. spinifera* were diagnosed by fungal culture and morphology alone [6,7]. One of the isolates was subsequently subjected to DNA sequencing of the ITS rDNA region (GenBank accession AF549450), which, similar to our case, confirmed the species identification [15].

The recommended treatment for localized feline cutaneous phaeohyphomycoses is surgical excision with wide margins, accompanied by systemic therapy with itraconazole or posaconazole pending antifungal susceptibility testing results [1]. The latter is important, since antifungal susceptibility profiles of dematiaceous fungi are variable. For example, high MICs of amphotericin B (>2 mg) have been found among *Exophiala* species [20]. Itraconazole therapy, alone or in combination with surgical excision, cryotherapy, terbinafine or minocycline was associated with partial or complete response in 16 of 20 cases of *E. spinifera* infections in humans [17]. Of the three previous *E. spinifera* infections in cats, outcome was known in one case of mucocutaneous nasal infection, in which surgical resection and therapy with ketoconazole and fluconazole was unsuccessful [7].

Gastrointestinal adverse effects to terbinafine and itraconazole are relatively common in cats. In this case vomiting continued despite discontinuation of terbinafine and may have been a gastrointestinal or hepatotoxic effect of the itraconazole. Itraconazole adverse reactions can be managed in some cases by dose reduction or by changing therapy to posaconazole [1].

## Declaration of competing interest

There are none.
